# Diagnostic utility of the neutrophil-platelet ratio as a novel marker of activity in patients with Ulcerative Colitis

**DOI:** 10.1371/journal.pone.0231988

**Published:** 2020-04-21

**Authors:** Jesús K. Yamamoto-Furusho, Erick A. Mendieta-Escalante

**Affiliations:** Department of Gastroenterology, Inflammatory Bowel Disease Clinic, National Institute of Medical Sciences and Nutrition Salvador Zubirán, Mexico City, Mexico; Kurume University School of Medicine, JAPAN

## Abstract

**Background:**

Ulcerative colitis (UC) is a chronic disease characterized by periods of activity and remission. The platelet, one of the main activators of neutrophils, contains Interleukin 8 (IL-8), a potent neutrophil chemo-attractant and P-selectin that induces excretion of superoxide in the neutrophils, forming platelet-neutrophil aggregates that are increased in individuals with active UC, hence an index of both cells could produce a monitoring tool. No previous studies have evaluated this ratio in UC.

**Goal:**

To evaluate the clinical utility of the Neutrophil-Platelet (NeuPla) ratio in patients with UC.

**Study:**

A total of 158 patients with a diagnosis of UC. This index was based on the ratio between platelets and the neutrophil differential in blood count. The activity was classified using Mayo endoscopic sub-score, histological (Riley score) and for clinical was used the Truelove-Witts, Montreal, Mayo and Yamamoto-Furusho scores.

**Results:**

The correlation of the NeuPla ratio with activity scales were significant (P <0.05). An optimal cut-off point to classify patients with clinical activity was 14.94 with a sensitivity 87.95% and specificity 63.5 and endoscopy activity with a cut-off 14.64 with a sensibility of 70.5% and specificity of 61.8%.

**Conclusions:**

The NeuPla ratio showed an adequate diagnostic utility to identify UC patients with clinical and endoscopy activity without the use of invasive studies like a colonoscopy or expensive fecal biomarkers such as calprotectin and have a better diagnostic performance in comparison to other serum biomarkers (C reactive protein, erythrocyte sedimentation rate and albumin).

## Introduction

Ulcerative Colitis (UC) is a disease under the spectrum of Inflammatory Bowel Disease (IBD) characterized by chronic inflammation in the colonic mucosa and submucosa with exacerbation (chronic diarrhea with blood, abdominal pain, fatigue, weight loss) and remission periods.

Biomarkers like erythrocyte sedimentation rate (ESR) and C reactive protein (CRP) are used for monitoring the disease but present a low specificity and sensitivity in UC [1]. There are fecal biomarkers with better performance for detecting disease activity in UC like fecal calprotectin and lactoferrin [2], however, are very expensive.

Previous studies have shown a strong relationship between neutrophils and platelets in the pathophysiology of UC. For example, histopathology findings are characterized by the presence of neutrophils in the colonic mucosa in the UC patient [3,4] and there are serological markers such as anti-neutrophil cytoplasmic antibodies (ANCAs) present in most of the UC patients [5]. From a biochemical aspect, the neutrophils are the principal source of calprotectin [6].

Platelets present granules with Interleukin 8 (IL-8) which is a potent neutrophil chemoattractant [7] and its cytoplasm contains P-selectin that induces overproduction of superoxide and the formation of neutrophil-platelet aggregates, which are elevated in patients with UC and related with the activity also in Dextran Sulfate Sodium (DSS)-Induced Colitis Model the inhibition of this aggregates suppress the appearance of colon inflammation [8,9]. The mean platelet volume (MPV) is correlated to the UC activity [10] as also the increase of white blood count (WBC) mainly neutrophils [11]. The interaction of neutrophil-platelet is a possible explanation of the high prevalence of thrombosis in UC due to the formation of Neutrophil Extracellular Traps (NETs) through Toll-like receptor 4 (TLR-4) [10]. A neutrophil-platelet ratio (NeuPla ratio) could be used as a monitor tool for UC, this ratio has been already used in other pathologies [12,13] and only required a complete blood count (CBC) making it an accessible, fast, easy to use and low-cost tool.

The present study explores the clinical utility of NeuPla ratio for the evaluation of UC activity and was correlated with endoscopy findings (Mayo sub-score, Montreal), histological (Riley score) [14], biochemical and clinical scores (Truelove-Witts and Mayo) [15,16] and novel integral index such as Yamamoto-Furusho score [17].

## Material and methods

**Fig 1:**
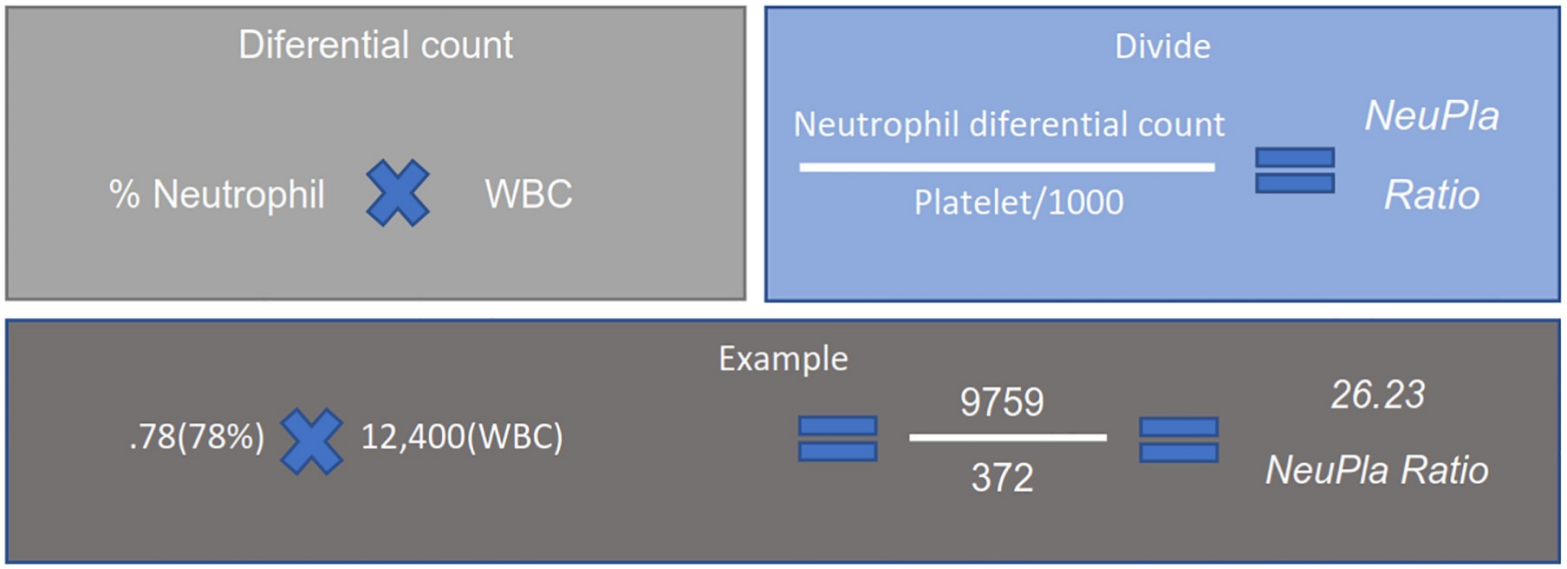
Procedure to calculate the NeuPla ratio. WBC; White Blood Count, NeuPla; Neutrophil-Platelet ratio.

A total of 158 patients with UC were included belonging to the Inflammatory Bowel Disease Clinic at the National Institute of Medical Sciences and Nutrition Salvador Zubirán (INCMNSZ) between July 2016 and September of 2019. All patients were older than eighteen years old with a definitive diagnosis based on clinical criteria (chronic diarrhea with blood), biochemistry, endoscopic features and histopathology findings. The clinical and demographic data were gathered from the clinical records: sex, current age, age at diagnosis, disease duration, extra-intestinal manifestations (EIMs), current medical treatment and extent of disease. Biochemical parameters included were CBC, CRP, ESR and fecal calprotectin. The NeuPla ratio was calculated by the differential count of neutrophils and the division with the platelets divided by 1000 as shown in Fig 1.

The biochemical activity was evaluated by fecal calprotectin, CRP and ESR. The endoscopy activity by Mayo sub-score and Montreal. Histological activity by Riley score, and clinical activity by Truelove-Witts, full Mayo score and Yamamoto-Furusho index. The full Mayo score was used to assess disease activity. Remission was defined as a full Mayo score ≤2. Mild activity was defined as a full Mayo score 3 to 5. Moderate activity was defined as a full Mayo score 6 to 9. Severe activity was defined as a full Mayo score 10 to 12.

The exclusion criteria were infectious colitis, lack of recent laboratory results, laboratory results from another hospital.

Results were expressed as mean ± standard deviation (or standard error of the mean) or median and interquartile range, depending on sample distribution. Comparisons between groups were made by Student’s T-test or U Mann Whitney for independent samples, according to the sample distribution. Spearman’s correlation coefficient was used for correlation. A curve ROC (receiver operating characteristic curve) for the optimal cut determining the sensitivity, specificity, positive predictive value, and negative predictive value. A p-value < 0.05 was considered statistically significant. The analysis was performed with SPSS software v. 24 (SPSS Inc., Chicago, IL).

### Ethical considerations

This work was accomplished according to the principles expressed in the Declaration of Helsinki. This study was approved by the research committee as well as the ethics of the National Institute of Medical Sciences and Nutrition Salvador Zubirán with reference number 3006 and also a written informed consent was obtained from all patients.

## Results and discussion

One hundred and fifty-eight patients were studied, and the clinical and demographic data are shown in Table 1. Serological and fecal biomarkers between patients in remission and clinical activity are shown in Table 2.

**Table 1:** Demographic and clinical characteristics of patients with UC.

Variables	
Sex (Male/Female)	73(44.5) /87(54.4)
Current age (years)	43.53±14.35
Age at diagnosis (years)	36.6±15.07
Years from diagnosis	11.27±8.136
**Activity**	
• Active	47(29.4)
• Remission	113(70.6)
**Extent of disease**	
• Proctitis (E1)	22(13.8)
• Left colon (E2)	31(19.4)
• Pancolitis (E3)	107 (66.8%
**Extraintestinal Manifestations (%)**	55(34.81)
• Arthralgia	28(17.1)
• Primary Sclerosing Cholangitis	12(7.3)
• Spondylitis	4(2.4)
**Treatment (%)**	
• Mesalazine	154(93.9)
• Steroids	43(26.2)
• Azathioprine	26(15.9)

Values are expressed as number (%), mean ± standard deviation

**Table 2:** Serological and fecal biomarkers in active and remission UC patients.

	Clinical Activity	Mean	Standard Deviation	P value
**Fecal Calprotectin**	REMISSION	397.2857	1092.37036	< .001
	ACTIVE	2163.2441	3316.19933	
**CRP**	REMISSION	.55419	1.037965	< .001
	ACTIVE	1.92998	3.380446	
**Albumin**	REMISSION	4.4519	.37880	< .001
	ACTIVE	4.0350	.55233	
**Platelet count**	REMISSION	267.39	71.619	.194
	ACTIVE	284.66	85.763	
**MPV**	REMISSION	8.472	.9252	.833
	ACTIVE	8.436	1.0969	
**WBC**	REMISSION	6115.32	1652.887	< .001
	ACTIVE	8454.23	2895.326	
**Leukocytes**	REMISSION	1833.78468	669.155945	.459
	ACTIVE	1748.09149	650.325310	
**Neutrophils**	REMISSION	3646.10435	1250.753429	< .001
	ACTIVE	5838.14843	2478.353209	
**NeuPla**	REMISSION	14.02382	4.529504	< .001
	ACTIVE	21.48726	8.950042	
**NLR**	REMISSION	2.21584	1.075825	< .001
	ACTIVE	4.06170	2.717627	

CRP; C Reactive Protein, MPV; Mean Platelet Volume, WBC; White blood count, ESR; Erythrocyte Sedimentation Rate, NeuPla; Neutrophil-Platelet ratio NLR; Neutrophil-Lymphocyte Ratio

### Correlation between NeuPla ratio and UC index scores

**Fig 2:**
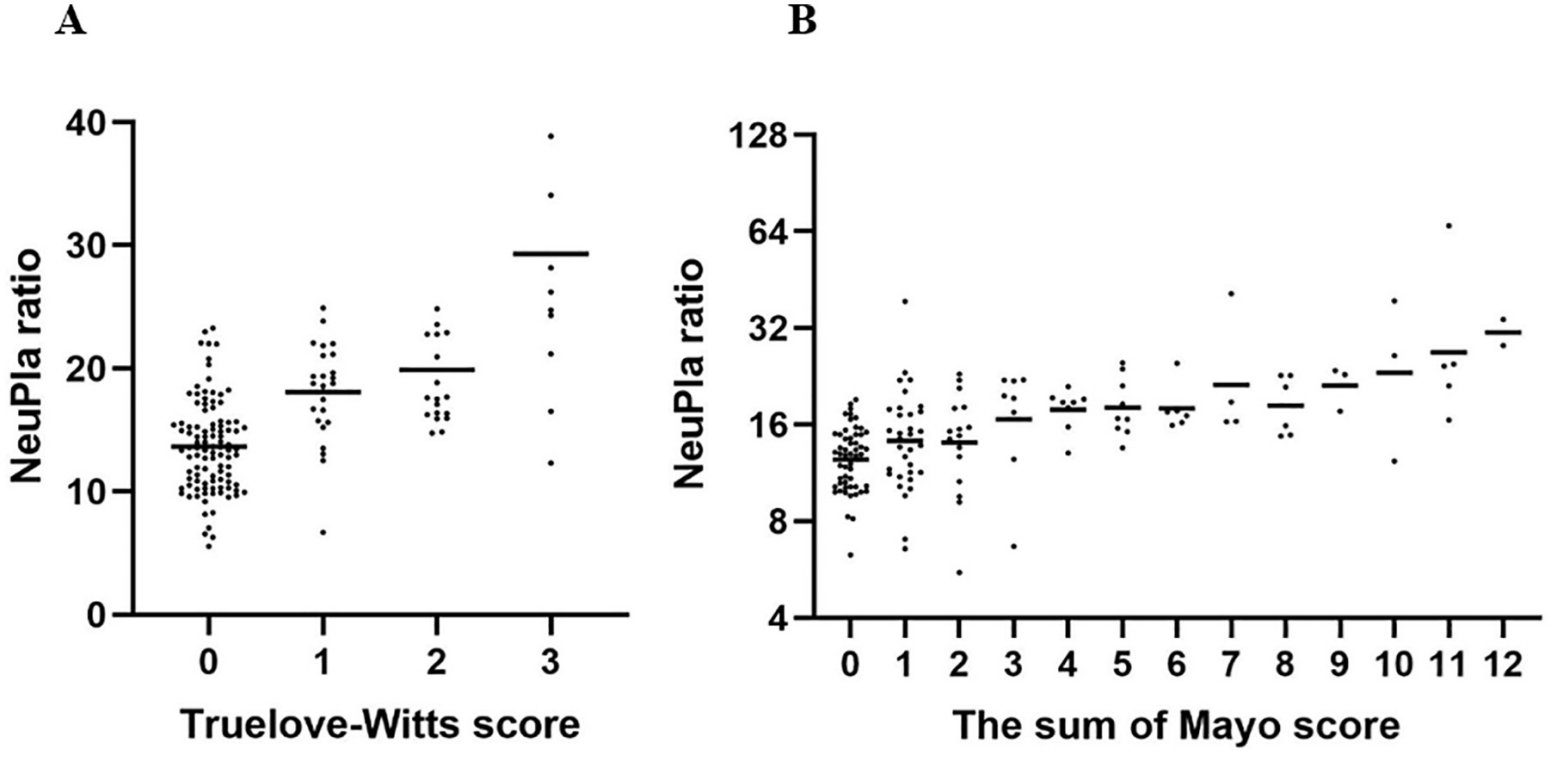
Distribution of NeuPla ratio. Scatter plot of NeuPla ratio and (A) Clinical activity (Truelove-Witts score); and (B) Clinical-endoscopic activity (Mayo full score).

The mean of the NeuPla ratio was 14.02 ± 4.52 in UC patients with remission, significantly lower compared to mild (16.4 ± 6.90 P = 0.003), moderate (18.39 ± 9.52 P = 0.0003), and severe activity (21.44 ± 5.39 P = 0.00003), the distribution of NeuPla ratio is shown in Fig 2. The correlation between the NeuPla ratio with several indexes was statistically significant for Mayo endoscopic sub-score, histopathological index (Riley score), Truelove-Witts, Yamamoto-Furusho, Full Mayo score, and Montreal as you can see in Table 3.

**Table 3:** Correlations of serological and fecal biomarkers with endoscopy, histology and activity scales.

		CALPROTECTIN	ENDOSCOPY	PATHOLOGY	TRUELOVE-WITTS	MAYO	YAMAMOTO-FURUSHO	MONTREAL
**NeuPla**	Rho	.532**	.462**	.288**	.476**	.585**	.544**	.346**
	P	.000	.000	.000	.000	.000	.000	.000
**NLR**	Rho	.347**	.310**	-.164**	.370**	.439**	.401**	.208**
	P	.000	.000	.000	.000	.000	.000	.000
**Calprotectin**	Rho	1.000	.658**	.363**	.567**	.691**	.608**	.519**
	P		.000	.000	.000	.000	.000	.000
**Neutrophil Count**	Rho	.453**	.482**	.285**	.469**	.573**	.607**	.377**
	P	.000	.000	.000	.000	.000	.000	.000
**Lymphocyte Count**	Rho	.111	.090	.020	.002	.045	.080	.137
	P	.265	.262	.805	.982	.574	.320	.085
**Platelet Count**	Rho	.081	.160*	.056	.136	.193*	.223	.179*
	P	.416	.045	.487	.088	.015	.003	.024
**CRP**	Rho	.330**	.355**	.205*	.294**	.348**	.613**	.295**
	P	.001	.000	.013	.000	.000	.000	.000
**ESR**	Rho	.195	.222**	.099	.196*	.273**	.315**	.156
	P	.051	.006	.234	.017	.001	.000	.058

CRP; C Reactive Protein, ESR; Erythrocyte Sedimentation Rate, NeuPla; Neutrophil-Platelet ratio NLR; Neutrophil-Lymphocyte Ratio

*<0.05

**<0.01

No association was found between the dose of corticosteroids more than 20 mg vs less than 20 mg (P = 0.51) and more than 20 mg vs non-users (P = 0.38). On the other hand, no association was found between standard dose of azathioprine vs low dose of azathioprine less than 125mg per day (P = 0.32) and vs non-users of azathioprine (P = .11).

### Correlation between NeuPla ratio and biochemical markers

NeuPla ratio surpasses the serum biomarkers correlations with endoscopy. As also, correlates with some of these biomarkers like CRP (Rho = .308, P = 0.0001), serum albumin (Rho = .383, P = 0.0001) and fecal calprotectin (rho: 0.532, P = .0001).

### Clinical utility of NeuPla Ratio

**Fig 3:**
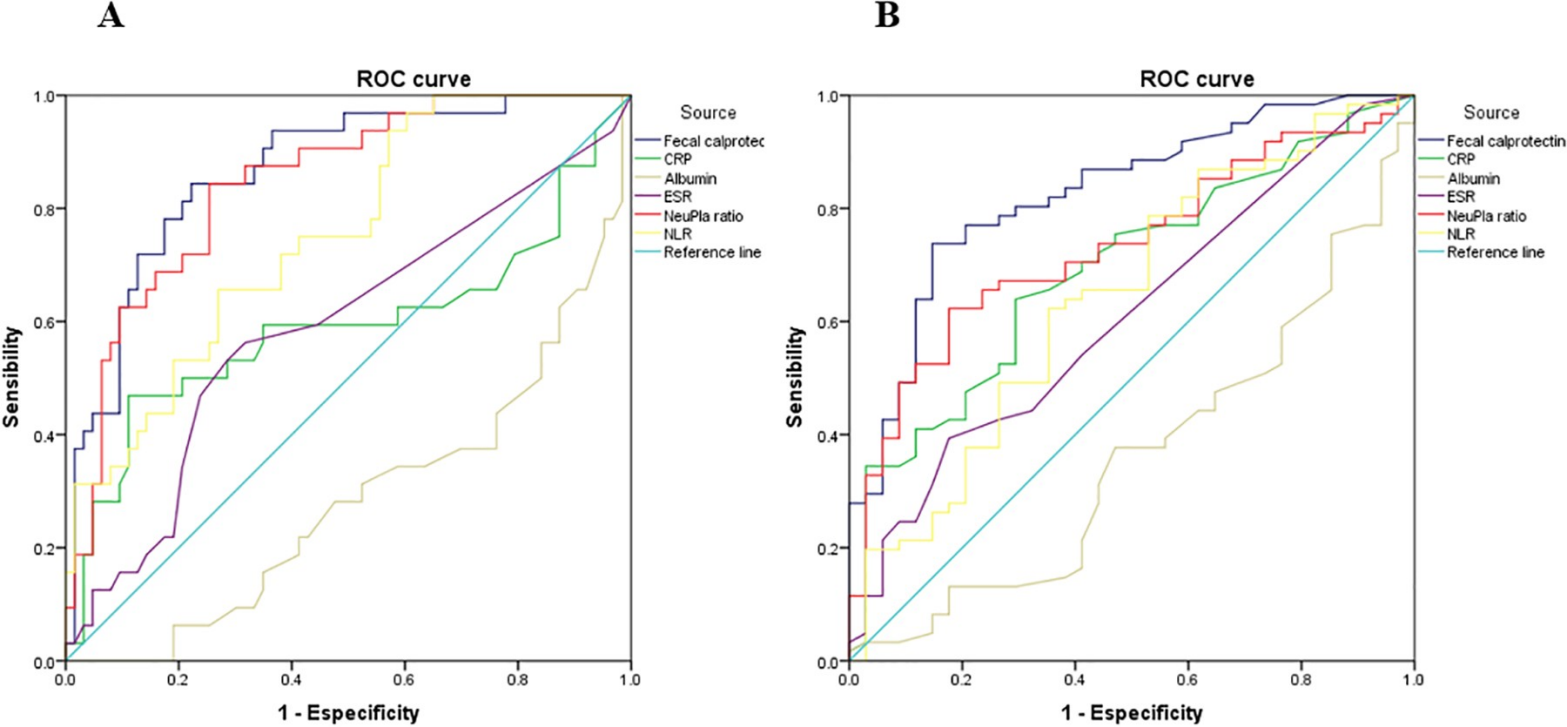
ROC curve of serological and fecal biomarkers to evaluate endoscopy activity. (A) ROC curve clinical activity; (B) ROC curve endoscopy activity. CPR; C Reactive Protein, ESR; Erythrocyte Sedimentation Rate, NeuPla; Neutrophil-Platelet ratio NLR; Neutrophil-Lymphocyte Ratio.

ROC curve was used to determine the cut-off level of NeuPla ratio according to clinical activity (optimal cut-off 14.94 with a sensibility of 87.95% and specificity of 63.5% and AUC of .853) as well as endoscopy activity (optimal cut-off 14.64 with a sensibility of 70.5%, specificity of 61.8% and AUC of .728) as you can see in Fig 3. The sensibility and specificity were made for other inflammatory biomarkers shown in Table 4.

**Table 4:** Diagnostic performance of the NeuPla ratio with other inflammatory biomarkers for the evaluation of endoscopic activity in patients with UC. Clinical and endoscopy activity.

Activity	Biomarker	Sensibility	Specificity
**Clinical**	CRP (>.355mg/dL)	59.4%	63.6%
	NLR (>2)	75%	52.4%
	NeuPla (>14.94)	87.95%	63.5%
	Fecal Calprotectin (>216)	90.6%	65.1%
**Endoscopy**	CRP (>.355 mg/dL)	52.5%	70.6%
	NLR (>2.09)	63.9%	58.8%
	NeuPla (14.64)	70.5%	61.8%
	Fecal Calprotectin (>366)	73.8%	85.3%

CRP; C Reactive Protein, NeuPla; Neutrophil-Platelet ratio NLR; Neutrophil-Lymphocyte Ratio

### Discussion

This is the first study to our best knowledge that evaluated the clinical utility of NeuPla ratio in patients with UC, the NeuPla ratio had a better diagnostic performance than traditional biomarkers such as CRP, ESR, and serum albumin with a sensibility and specificity similar to fecal calprotectin, however, NeuPla ratio has a lower cost, easy access and faster results because it can be calculated with a complete blood count. Also, is adaptable for hospitals that are lacking of fecal calprotectin or low access due to the high cost.

Many studies demonstrated a clinical performance of Neutrophil-to-Lymphocyte Ratio (NLR) in UC [18,19] though some of them showed a suboptimal function and low correlation as also with no association with disease extension [20,21]. It had been suggested in NLR that the prognostic value could be produced by the total count of neutrophils and lymphocytes count makes little contribution or neither [22]. NeuPla ratio had a higher correlation with disease activity a better sensibility and specificity as also correlated with extent of disease (rho = .346 P = < .05) making it a better tool for UC. An explain of the better performance could be seen in Table 3 as platelet count (one of the elements of NeuPla ratio) had a better correlation with endoscopy activity, Full Mayo and Montreal scores than lymphocyte count (one of the elements of NLR).

The functionality of NeuPla ratio in UC might be explained by neutrophil-platelet relation is important in the process for migration of neutrophils to the tissues and contributes to the inflammation process [23]. On the other hand, Crohn´s disease (CD) has a delay of the recruitment of neutrophils to sites of infection and trauma as also an abnormally low secretion of IL-8 (potent neutrophil chemo-attractant) [24,25]. Some studies had been taken the neutrophil-platelet approach. For example a study on colorectal cancer and neutrophil-platelet score (NPS) showed that highest NPS score present a worse outcome as also high correlation with CRP and serum albumin the authors hypothesized that this score could be an inflammatory systemic marker and that had also clinical utility in other types of cancer like breast, bladder, and prostate [22]. In the ischemic stroke, it is well taken as a survival marker to 90 days as it has been demonstrated that a lower NeuPla ratio translates as less inflammation and necrosis due to authors conclude again that the NeuPla ratio is a tool that could measure inflammation and necrosis [12].

The importance of this study recalls in showing the neutrophil and platelet could be also a piece of the pathogenesis in the inflammatory process in UC patients.

NeuPla ratio is a way to show the proportion between neutrophils and platelets a NeuPla ratio of 10 means that are 1 neutrophil per 100 platelets. This relation rises when a flare or activity of the disease appears, may be because as proportion more neutrophils are produced than platelets or because the platelets take 2 to 4 days to rise even 6 in their active form [26]. This UC population has no presented platelet count more than 450,000 cells per microliter defined as thrombocytosis and it could be possible that patients who present a severe activity, the NeuPla ratio is not useful because the clinical symptoms are obvious. However, NeuPla ratio could be very useful in those patients with mild to moderate activity in which the symptoms are not evident in order to optimize the medical treatment and avoiding complications or a bad outcome. Finally, NeuPla ratio could be used in hospitals where lack of fecal biomarkers like calprotectin or limited access for the cost and time for getting results.

In conclusion, the NeuPla ratio showed an adequate diagnostic utility to identify patients with clinical and endoscopy activity in UC patients without the use of invasive studies like a colonoscopy or expensive fecal biomarkers such as calprotectin. It is necessary to perform future studies to understand the nature of this Neutrophil-Platelet proportion in UC patients.

## Supporting information

S1 Data (XLSX)
